# Construction Notes

**Published:** 1894-08-04

**Authors:** 


### CONSTRUCTION NOTES.

### Smallpox Hospital for Dundee.

The site selected for the accommodation of small-pox patients by the Dundee Police Commissioners is on the lands of Baldovan, lying outside the borough. The ground, which extends to about seven acres, has been enclosed, and it is regarded as eminently suitable for the purpose to which it is put. The buildings consist of four blocks, and will cost £5,666, exclusive of any additional work that may have yet to be gone into. The first block comprises the porter's lodge, which contains a room for the porter, with kitchen, parlour, and bedroom, along with a waiting-room for visitors or inquirers. Two stories in height, the administrative block contains on the ground-floor a kitchen, scullery, nurses' dining-room, nurses' sitting-room, dispensary, and two large stores; while the upper floor is divided into six bedrooms for nurses, a bath-room, &c. The block stands some distance in front of the small-pox hospital, both leading from it in an open corridor to the small-pox pavilion, which forms the third block. The last-mentioned building has accommodation for ten male and ten female patients, in addition to which are two nurses' rooms, scullery, store-rooms, bath-room, and other conveniences. The pavilion is limited to one story, but is so arranged that it can be readily extended, and indeed there is room for the erection at least of two other pavilions. The fourth block will occupy a site central between the pavilion and that of the site suggested for a cholera hospital, and it will consist of laundry, offices, &c. These will contain wash-house, laundry, and drying closet, engine-room, mortuary, boiler, disinfectors, &c. The mason work contract was accepted from Messrs. A. T. Craig; laundry machinery from Messrs. Bradford and Co., Manchester. Disinfectors were supplied by Messrs. Manlove, of Nottingham. The Borough Engineer had the drawing-up of the architectural plans. The commissioners further decided to lay down a permanent floor for a temporary hospital for cholera patients on an adjacent site. A remit was made to Mr. MacKinson, Borough Engineer, to prepare plans and specifications for the erection of the permanent hospital. Meanwhile the work which it has been arranged shall now be proceeded with will involve an expenditure of £770, of which sum £320 will be expended on the floor. The building is designed to admit twelve patients.

